# Extrusion 3D Printing of Polybutyrate-Adipate-Terephthalate-Polymer Composites in the Pellet Form

**DOI:** 10.3390/polym10080922

**Published:** 2018-08-17

**Authors:** Sarat Singamneni, Dawn Smith, Marie-Joo LeGuen, Derryn Truong

**Affiliations:** 1 Department of Mechanical Engineering, Auckland University of Technology, Auckland 0632, New Zealand; derryn.truong@hotmail.com; 2 Scion Research, Rotorua 3010, New Zealand; Dawn.Smith@scionresearch.com (D.S.); MarieJoo.LeGuen@scionresearch.com (M.-J.L.)

**Keywords:** 3D printing, fused deposition modelling, polybutyrate, PBAT, wood flour composite, pellets

## Abstract

Fused deposition modelling is a common 3D printing technique used for the freeform fabrication of complex shapes based on polymers. Acrylonitrile butadiene styrene (ABS) is the common material option, though polylactide (PLA) has also proved to be a successful candidate. There is an ever increasing demand to harness new materials as possible candidates for fused deposition. The current research is focused on evaluating polybutyrate-adipate-terephthalate–polymer (PBAT) for fused deposition modelling. Both neat and composite PBAT filled with varying wood flour fillers were experimentally analyzed for 3D printing by extrusion from the pellet forms. The results are positive and the addition of small quantities of the wood flour filler material was found to improve the thixotropic nature of the polymer composite and consequently the inter-strand and inter-layer coalescence.

## 1. Introduction

Fused deposition modelling is an extrusion 3D printing process, mostly based on polymers in the filament form. However, it can also use the raw feedstock materials in the pellet form, which drastically reduces the material costs, as the production of a uniform filament is time consuming and relatively expensive. However, not all polymeric materials are suitable for processing from the pellet form. In this paper a biopolymer composite based on polybutyrate-adipate-terephthalate–polymer (PBAT) or polybutyrate is evaluated for possible use as a raw material in the pellet form for processing by extrusion 3D printing. PBAT is copolymer made up of adipic acid, butanediol, and terephthalic acid and though synthetic, is biodegradable [1]. Terephthalic acid gives rise to high thermal stability and mechanical properties while adipic acid and butanediol impart flexibility and biodegradability.

Biopolymer composite films made by polylactide (PLA)/PBAT blends filled with finely ground Babassu powders were analyzed by Franca et al. [1] as alternatives for cost effective packing solutions. Evaluation of specimens made by solvent casting techniques showed acceptable mechanical properties but evidence of immiscibility in the Fourier-transform infrared spectroscopy (FTIR) and differential scanning calorimetry (DSC) results. An addition of 10% of Babassu to the PLA/PBAT blend was found to be the most optimum and cost-effective solution. Polymer composites based on PBAT and cellulose nanocrystals were analyzed by small amplitude oscillatory shear experiments showing that the elastic behavior enhances with increasing amounts of the nano-cellulose, revealing a reinforcing effect of the nano particles [2]. Spatial reorganization of the nano-rods was observed further to a thermal annealing treatment at 170 °C for 30 min. Polybutyrate is a flexible and tough biodegradable polymer and in the current study, wood flour will be used as the filler material while extrusion 3D printing from the pellet form.

Cicala et al. developed Polythermide (PEI) blends based on either polycarbonate (PC) or polyethylene terephthalate glycol-modified (PETG) and evaluated the same for processing by fused deposition modelling in comparison with the commercial Ultem 9085 material option by Stratasys [3]. Based on rheological, morphological, and thermomechanical responses, the PEI/PC blends with 10% by weight of the modifier were proved to exhibit viscous behavior close to the commercial Ultem material, while PEI blends with PC contents lower than 20% by weight were found to be better than the commercial Ultem in terms of thermomechanical responses. Cicala et al. also evaluated three commercial grade PLA filaments in terms of the quality of printing a complex shape with overhang features [4]. Evaluations based on shear thinning studies, scanning electron microscope (SEM), and thermo-gravimetric analysis proved that the filaments with mineral fillers result in the best printing quality.

Matsuzaki et al. experimented composite fiber materials infused into PLA for 3D printing, targeting better mechanical properties for the printed parts [5]. A FDM printer was modified in order to impregnate the filament with composite fibers during extrusion. The reinforcing fiber was heated using a nichrome wire before it entered the nozzle to enhance the permeation of the fiber bundles with the thermoplastic resin. The resin filament is melted by the heater inside the nozzle, consolidating the reinforcing fibers and the resin in the heating chamber. Final results show superior Young’s modulus and strengths compared with materials fabricated using commercial 3D printers. Guo et al. also adopted wood-plastic composites for selective laser sintering (SLS) [6]. The main composition consisted of wood flour and a co-polyester powder that accounted for over 90% of the mass. The overall void fraction was obtained to be 51%, prior to post processing. Wax infiltration was shown to reduce the void fraction to 7%. The final components produced were reported to have relatively high dimensional accuracy with sufficient strength.

There was little evidence of PBAT processed by additive technologies in the past, but other conventional methods were attempted. Ludvik et al. investigated the addition of cellulose fibers in PBAT with bentonite clay to improve fiber dispersion and result in a higher water resistance [7]. The average modulus of the composite material was reported to be higher than that of the composition without the cellulose fiber. However, the strength and elongation at break displayed the opposite relationship, which could potentially be overcome by surface modifications of the fiber-matrix. It was noted that weak adhesion between the cellulose fiber and the thermoplastic/clay matrix after the drying stage could cause the fiber to clump during processing, unless a special binder was implemented. Yeh et al. studied the compatibility, crystallization, and tensile properties of PLA/PBAT blends in different proportions produced by melt blending [8]. The results showed PBAT to be effective in increasing the toughness of PLA.

The focus of this article is to establish the feasibility of the combination PBAT-wood flour blends to be processed by extrusion 3D printing and establishing the ideal process conditions for the best quality of the printed samples. The emphasis is on the conjoined attributes possible by combining PBAT and wood flour, a biodegradable polymer fibrous material. Blends of the two constituents in different compositions will be evaluated in the pellet form for extrusion 3D printing with varying process conditions.

## 2. Materials and Methodology

### 2.1. Materials

The biodegrade material under investigation in this paper is PBAT with different wood flour compositions as listed in Table 1 infused into the base polymer. The polymer composites are developed and compounded by twin screw extrusion at Scion (Rotorua, New Zealand). The extrusion was carried out on a 26 mm co-rotating twin-screw extruder (Labtech Engineering Ltd, Praksa, Muang, Samutprakarn, Thailand) with a L/D ratio of 40. The compositions of wood flour used include 0 wt % (Neat), 5 wt %, 10 wt %, and 20 wt %.

### 2.2. Basic Print Parameters

The melting temperature of the material was identified to be 120 °C from the data sheet of ecoflex^®^ F Blend C1200. The material was preheated at 50 °C for 15 min using an electric oven, to remove residual moisture that may have accumulated [9]. A homemade extrusion 3D printer developed at AUT was used in these experimental trials [10]. The extrusion chamber was also preheated to the specified melting temperature of the material (120 °C), allowing time for the chamber to reach a steady state temperature and enabling a consistent heat transfer into the polymer to melt progressively and attain a uniform flow.

**Figure 1:**
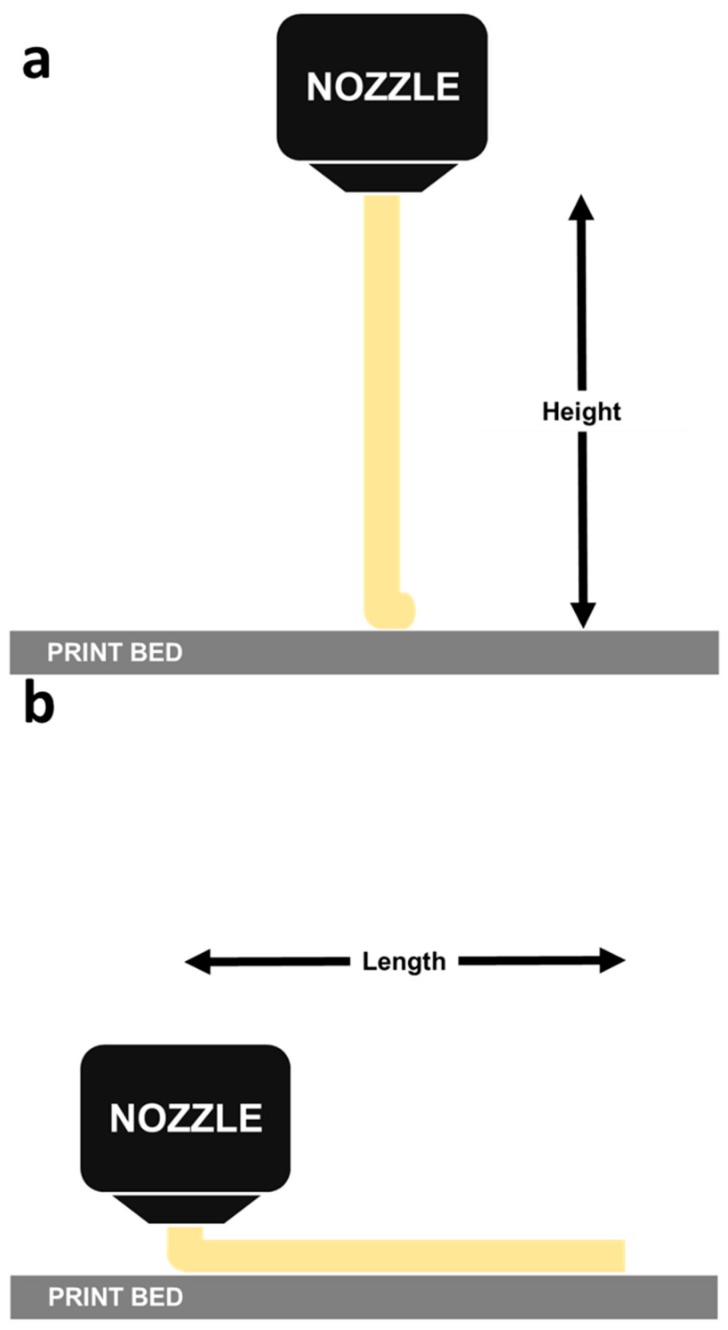
Evaluation of (**a**) the extrusion and (**b**) print velocities.

Mesostructures resulting from the constrained coalescence between strands [11] and certain process parameters [12] were reported to be critical in controlling the quality of parts produced by fused deposition modelling. For consistent material deposition and consolidation, the extrusion (spindle speed) and print velocities should be synchronized. This is to ensure no build-up of excess material (bulging) due to a faster extrusion rate with a slower printing speed. A slower extrusion rate with a high printing speed would cause the filament to be stretched, which is also undesired. To identify the extrusion velocity, the nozzle was positioned at a fixed vertical height (200 mm) from the print bed. Using initial spindle speed of 1300 RPM, the time taken for a filament to travel the height was recorded, from which the experimental extrusion velocity could be calculated, as illustrated in Figure 1a. This height must be relatively small as at elevated distances, gravitational forces would have a more significant impact on the end result. To identify the print velocity, the nozzle was positioned at a fixed horizontal distance (200 mm) from one end of the print bed, the time taken for the nozzle to travel the fixed distance as shown in Figure 1b was recorded. The experiment was repeated with different distances and the data generated was used to establish the printing and extrusion velocities as 1300 RPM and 540 mm/min respectively. Multiple trials were conducted to ensure reliable data generation.

### 2.3. Critical Temperature Settings

Printing trials began first with the base polymer, PBAT100 and then extended to the polymer composites with increasing wood flour content, PBAT95, PBAT90, and PBAT80. Once the printer is prepared and after reaching the desired temperatures, the heated polymer pellets were slowly fed into the hopper of the FDM printer. As the pellets move through the length of the feed chamber the temperature gradually rises and finally the plasticized pool of polymer gets collected in the extrusion chamber. Due to the pressure building up from the continuous accumulation, the semi-solid material is extruded from the nozzle tip (2 mm diameter) onto the print bed. Initially, the extrusion and bed temperatures were set to 110 °C and 90 °C respectively, as identified from the data sheet. However, the extrusion temperature at 110 °C proved to be insufficient, as the polymer was sticking to the surfaces and causing problems to the screw feeding. As a result, the temperature of the extruder was increased by 5 °C intervals until a continuous filament was produced, eventually at 125 °C.

### 2.4. Filament Settings

The diameter of the extruded polymer filament was found to increase with prolonged exposure to moisture, with a certain absorption rate. The moisture would cause aeration during extrusion, as the water vaporizes at high temperatures, air bubbles form within the filament resulting in an increase in the diameter. It would also weaken the overall structure with the addition of internal porosities. To remove the residual moisture that may have accumulated from the environment, the material was preheated in an oven at 60 °C for 15 min prior to printing.

Ideally, during extrusion, the viscosity should be low enough for continuous flow at high temperatures, but not too low that the flow behavior is non-uniform. However, during printing, the viscosity should be high enough that it is able to promote sintering, without drastic variations in the shape of the polymer strand [13]. The ability of the polymer to maintain an appropriate viscosity value throughout the various stages of printing dictates the quality of the polymer sintering, which has a direct impact on the mechanical properties of the printed components.

Using the optimized extrusion temperature, a continuous strand was made to observe the printing behavior and consistency. The distance between the nozzle tip and the print bed (air gap) was determined by visual examination, to accommodate the size variation of the filament with an additional gap to provide clearance between the part and the nozzle, to avoid physical contact. The distance between each subsequent strand (strand gap) was also determined visually. A small overlap of about 10% between adjacent strands was included to promote inter-strand sintering based on the print quality at the best extrusion temperature identified, 125 °C. The next step was to print single layer rectangular samples (20 × 50 mm) to observe the sintering behavior on a preliminary level. Multi-layer samples (4 mm thick) were then printed to examine how the parameters interact when printing three dimensionally.

## 3. Initial Experimental Results

### 3.1. Optimal Printing Parameters

The optimal settings for the critical printing parameters; extrusion temperature, bed temperature, extrusion velocity (spindle speed), print velocity, strand gap, and air gap were identified mainly based on visual inspection during the initial printing process. The parameter levels identified are as listed in Table 2. It is important that the extruded filament remains in a semi-solid state, where the material has a moderately high viscosity and will retain its cylindrical shape [11,12,13,14,15,16]. This is to help facilitate interlayer sintering between neighboring strands. If the filament is either in a solid or liquid state, the component will not sinter properly or have immense dimensional variations respectively. The nozzle and chamber were both exposed to the atmospheric conditions, hence the actual temperature inside the chamber may be lower than the indicated temperature. This would explain the discrepancy between the specified melting temperature from the data sheet and the actual extrusion temperature, which was caused by heat loss from the chamber. The heating element is located only near the bottom of the nozzle.

The base platform is heated, otherwise the sample will be cooled prematurely causing it to shrink and lift off the platform. This issue was problematic during multilayer samples, as the heat from the platform is not sufficiently able to be transferred through the multiple layers. This caused the sample to lift off the platform during the printing of another layer. Increasing the base temperature causes the polymer to melt as the base temperature approaches the melting temperature. To resolve this problem, masking tape was applied to the platform for additional surface resistance and frictional forces. The heated temperature should be just below the glass transition temperature to keep the filament in a semi-solid state (110 °C). The glass transition temperature is mainly associated with amorphous polymers, which is the temperature that provides sufficient energy for the ions to move without restriction. It was difficult to maintain this temperature with the multilayer samples, as the print chamber is not completely enclosed.

### 3.2. Strand Continuity

**Figure 2:**
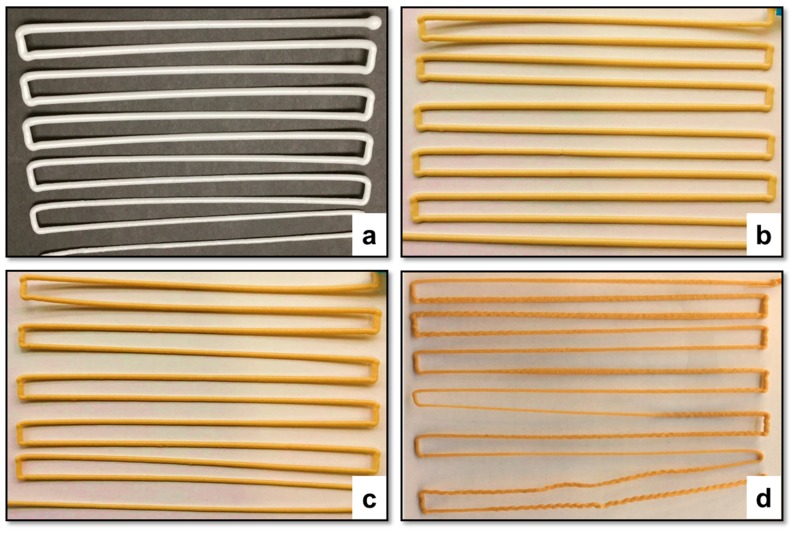
Images of continuous filaments printed using (**a**) PBAT100, (**b**) PBAT95, (**c**) PBAT90 and (**d**) PBAT80.

Pellet-based extrusion 3D printing using polymer composites loaded with filler materials often gives rise to problems in the continuity of the extruded filament. Experiments based on continuously printing the filament were conducted to observe the dimensional stability of the strand generated as shown in Figure 2. A material with a good stability during extrusion would have almost a constant filament diameter with minimal variation. As seen in Figure 2, PBAT95 (b) and PBAT90 (c), produced the most uniform filament strands, while the diameter of PBAT100 (a) continued to decrease gradually. PBAT80 (d) had an unusual result, where the filament diameter was continuously changing somewhat arbitrarily and at certain points the filament became wavy.

The pure PBAT and the PBAT-wood flour composites at higher filler content behaved in similar ways in terms of the filament consistency. With time, the polymer develops blockage through the nozzle, either due to the accumulation of the partly solidified mass in the case of pure PBAT or the filler material in the case of the PBAT-wood-floor composites with excessive filler materials such as the PBAT80 in this case. In between, the other two cases at 5% and 10% filler contents showed the best extrusion characteristics of the polymer composite. Clearly, small quantities of the wood-flour when added to the base polymer help improve the extrusion responses of the composite. However, beyond 10 percent by weight, solid wood particles aggregate in the nozzle and destabilize the extruded filament characteristics and consequently the printing process.

### 3.3. Single Layer Samples

**Figure 3:**
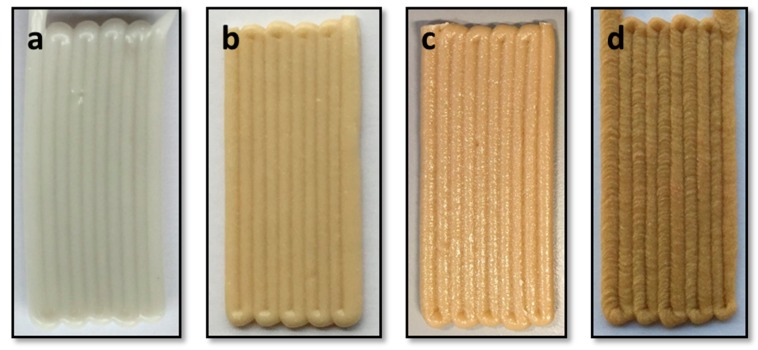
Single samples of (**a**) PBAT100, (**b**) PBAT95, (**c**) PBAT90 and (**d**) PBAT80.

Single layer samples (20 × 50 mm) were printed to observe the sintering behavior at a preliminary level. All the materials could be printed as single layer samples to a high degree of quality as may be noted in Figure 3, by adjusting the process parameters as listed in Table 2.

### 3.4. Multilayer Samples

**Figure 4:**
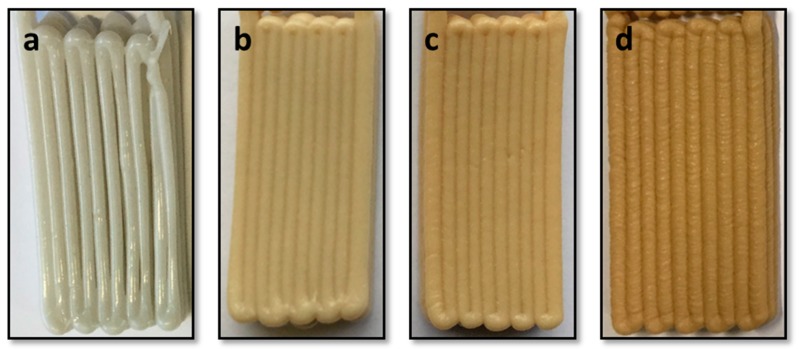
Multilayer samples of (**a**) PBAT100, (**b**) PBAT95, (**c**) PBAT90 and (**d**) PBAT80.

Multi-layer samples with 3 layers (6 mm in thickness) were printed to examine how the parameters interact while achieving the inter-layer coalescence. As shown in Figure 4a, PBAT100 had difficulties when printing multiple layers. This is due to a gradual decrease in the filament diameter as may be observed in Figure 3a. The first two layers of the sample could be printed successfully, but, during the final layer, the material flow began depleting. The neighboring filament strands would not come into contact with each other, leading to a clear lack of inter-strand coalescence. The other three samples with varying amounts of the filler material showed clear evidence of improved consolidation. In particular, samples with 5% and 10% wood contents gave the best results in terms of the inter-strand coalescence. With further increase in the wood-flour content, the polymer loses some of the thixotropic nature, leading to premature solidification and lack of coalescence between adjacent strands.

The dimensional variation of the overall sample was evaluated using digital caliper measurements central to the length of the sample [17]. As per the CAD model, the dimensions of the samples should be 6 mm in thickness and 20 mm in width. The results presented in Table 3, show that both thickness and width increased, which is probably due to the aeration and expansion of the filament upon exit from the nozzle. Relatively, the thickness of the three layered samples decreased with the amount of wood flour content, while the width of the sample increased. It was also noted that the thickness of PBAT80 was below the designed value, whereas the other material compositions resulted in measurements above the theoretical values. This may have been caused by the elevated extrusion temperature, resulting in a loss of viscosity of the material and the ‘running away’ of the relatively soft substrate. This is also reflected in the width of PBAT80, where it substantially increased compared to the other samples.

## 4. Further Experimentation and Results

### 4.1. Smaller PBAT100 Samples

**Figure 5:**
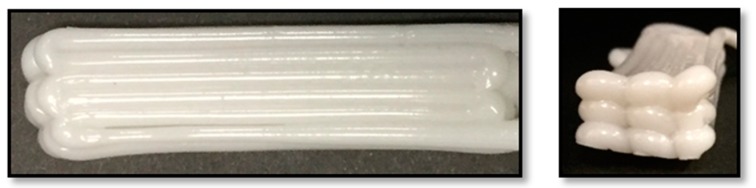
Smaller sample of PBAT100.

It was noticed from Figure 2 that the dimensional variation of PBAT100 displayed a trend of continuous decrease in the filament diameter. However, during the initial printing (between 0–600 mm), the filament has a relatively stable diameter size. To observe the sintering responses of the base polymer (PBAT), smaller multilayer samples (10 × 50) of PBAT100 were printed as shown in Figure 5, which were able to be printed successfully and to a high degree of quality, based on visual inspection. However, as larger samples were not able to be printed, due to the difficulties associated with the extrusion, further investigation of PBAT100 was not conducted.

### 4.2. Intermediate Cleaning of Nozzle

**Figure 6:**
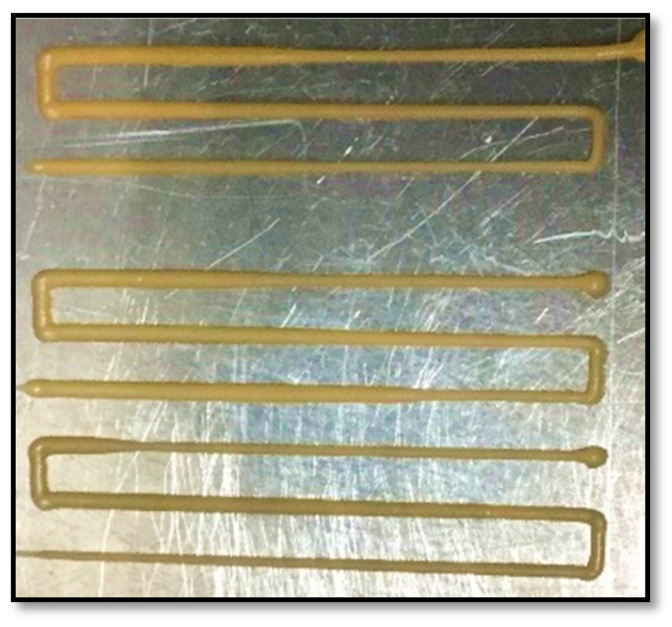
Printing with intermittent cleaning.

The reason behind the diameter inconsistency is due to the coagulation of the wood polymer particles during extrusion, where the denser wood particles are clogged somewhere in the nozzle, while only the softer polymer gets extruded. In an attempt to mitigate this effect, the printer was paused after half the sample was printed and a sharp needle was inserted into the tip of the nozzle to push the clogged wood particles out. This intermediate cleaning of the nozzle should relieve the built-up pressure of the wood particles, creating an unencumbered path for the filament flow. The results were slightly successful and displayed some improvements in the flow consistency as shown in Figure 6. However, since the material is pushed upwards by the needle, during the resumption of the printing, very little material was deposited initially, requiring some time for the material to regain its flow. Ideally, the extrusion process has to halt after every couple of layers, and the extrusion head needs a clean-up at the home position, which is the normal procedure adopted in any commercial system.

### 4.3. Addition of Stainless Steel Powder

PBAT100 experienced the maximum difficulty during extrusion, as the filament diameter gradually decreased over time. The possible reason for this is perhaps a lack of mechanical driving force for the mass transport. With PBAT100, being highly viscous when heated and softened, gets stuck near the nozzle, and creates an air gap due to back pressure. However, filler material added to the base polymer was found to enhance the mechanical forces, allowing for better extrusion. With the extrusion temperatures employed, the wood flour is not melted and remains in the solid state. The inter-particle frictional forces generated will probably break some of the viscous bonds, leading to a better mass transport phenomenon along the length of the extrusion chamber and out of the nozzle tip.

**Figure 7:**
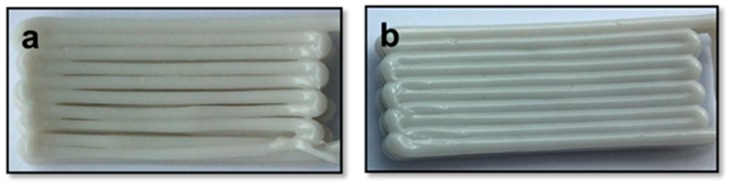
Photographs of (**a**) PBAT100 and (**b**) PBAT100 with stainless steel powder.

To examine this proposition, a 5 wt % of 30 μm of stainless steel powder was mixed with the PBAT100 pellets, prior to printing. This experiment is to verify the observation that the excess force is due to the solid-state particles infused into the plasticized polymer composite material. Theoretically, stainless steel is denser than PBAT, which should significantly boost the flow rate of the mixture. Observing the images of the printed samples as shown in Figure 7, a great deal of improvement may be noticed. A multilayer 20 × 50 mm sample was able to be printed with the addition of the stainless steel powder (b), where it was previously almost impossible to work with just PBAT100 (a).

### 4.4. Injection Moulding

**Figure 8:**
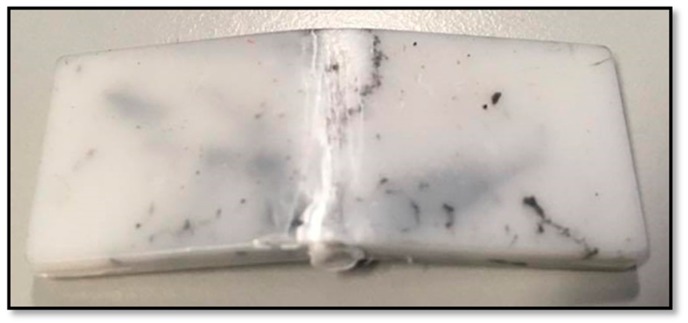
Injection molded sample of PBAT100 with stainless steel powder.

Injection molded samples of the same materials were produced for a comparative evaluation of the mechanical properties of samples produced by the two manufacturing methods. The pure PBAT injection molding is shown in Figure 8, and the shaded colors are due to some residual material in the molding setup from a previous trial. The extrusion chamber of the machine was not able to be fully cleaned. A comparison of the mechanical properties of the 3D printed (3DP) and injection molded (IM) samples was done to assess the ideal characteristics of the material under 100% coalescence. From Table 4, it can be seen that the flexural modulus of the injection molded samples was significantly greater than that of the 3D printed counterparts, which is common with fused deposition modelling [18]. However, the maximum bending stress values show a closer correlation between the 3D printed and injection molded samples.

## 5. Discussion

### 5.1. Filament Consistency

**Figure 9:**
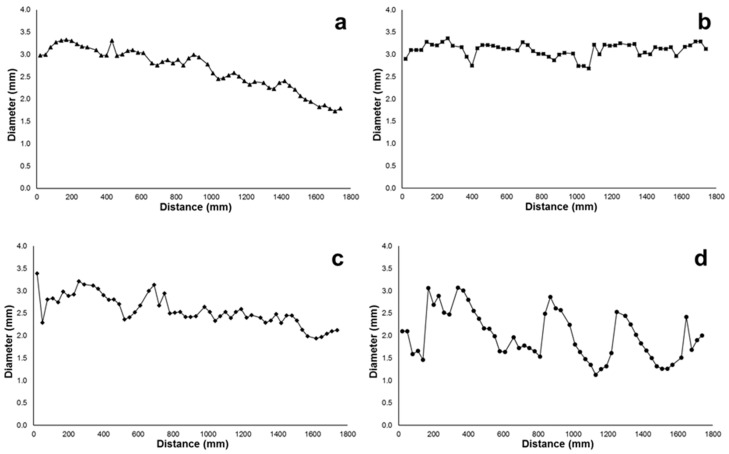
Thickness variation of continuously printed samples of (**a**) PBAT100, (**b**) PBAT95, (**c**) PBAT90 and (**d**) PBAT80.

The diameter of the continuously printed strand was measured at 30 mm intervals to identify trends for an analytical representation. The diameter of the filament produced by extruding PBAT100 displays a continuous decline in the measured values, with no indication of any stabilization at the end. There are also a few random peaks and troughs as illustrated in Figure 9a. The probable causes of this are poor thermal characteristics or premature solidification. With PBAT95, a more consistent filament could be produced, with minimal variations, as seen in Figure 9b. This is interesting as the addition of the wood flour mitigated the filament size inconsistencies.

It is envisioned that the addition of the filler material aided in the mechanical forces generated to help extrude the material, probably due to a change in the viscosity. PBAT90 displays a gradual decrease in the thickness trend similar to PBAT100, but to a lesser degree and with a greater number of random spikes in the diameter as shown in Figure 9c. This may be attributed to the relatively higher level of accumulation of wood flour particles in the nozzle head. The diameter of PBAT80 was unstable in that the flow was inconsistent and would fluctuate between significantly higher and lower values. This was difficult to control using the printing parameters, while the only parameter that affected the quality of the strand was the extrusion temperature. The higher the temperature, the better the consistency, but increasing the temperature too high would result in a low viscosity strand that flattens due to its own weight, which explains the average of 2 mm thickness shown in Figure 9d.

### 5.2. Mechanical Properties

**Figure 10:**
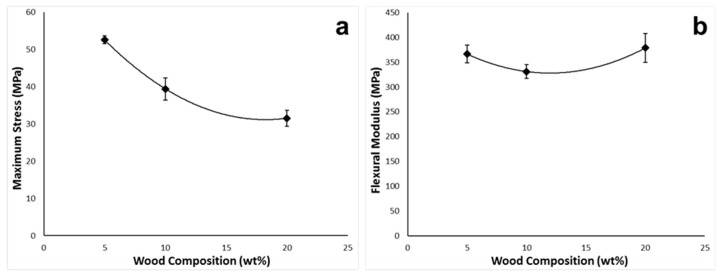
(**a**) Stress versus composition, (**b**) flexural modulus versus composition.

Results of the 3-point bending tests conducted on the multi-layer specimens are presented in Table 5. It was observed that the force required to deform the PBAT80 sample was significantly lower than that with the other materials. This is probably caused by the poor sintering quality of the sample due to the fluctuating diameter of the extruded filament. This is also reflected in the variation of the maximum strength of the material, as depicted in Figure 10a as the maximum stress value decreased with increasing wood flour content. The flexural modulus results, however, show only a slight variation as depicted in Figure 10b. The ductility of the polymer decreased with increasing wood-flour as the filler material makes the polymer stiffer, offering resistance to the unfolding and elongation of chains.

### 5.3. Optical Microscopy

**Figure 11:**
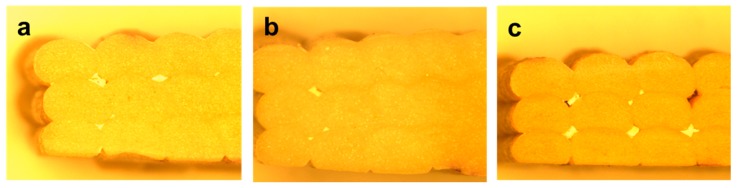
Optical microscopy images (6.3× magnification) of (**a**) PBAT95, (**b**) PBAT90 and (**c**) PBAT80.

Cross-sections of the printed samples were placed under an optical microscope and images taken at 6.3 times magnification are presented in Figure 11 to evaluate the inter-strand and inter-layer coalescence. The coalescence value is used as an indicator of the density of printed samples and the resulting meso-structures. The results show that PBAT95 had the best overall coalescence at 98%, while there is a gradual loss of the same with increasing wood flour content, resulting in 82% and 64% coalescence levels with PBAT90 and PBAT80, respectively. This is also in accordance with the filament consistency results, proving that the dimensional variation has a direct effect on the meso-structural quality of the printed material.

## 6. Conclusions

PBAT and food-flour composites are evaluated for 3D printing by extruding from the pellet form. The results are generally positive, indicating the suitability of the polymer composite for 3D printing directly from pellet forms. However, the mechanical properties of the printed parts are poor relative to the injection molded counterparts, though it is always the case with any printed object, considering the meso-structural lapses. Pure PBAT exhibited lack of filament consistencies and consequent loss of print quality. Adding filler materials up to a certain extent was proved to improve the extrusion characteristics significantly, which has also reflected in the enhanced print quality. The maximum strength of the printed parts gradually decreased with increasing wood filler content. Samples printed extruding the 95% PBAT and 5% wood-flour filler resulted in the optimum response levels at the best coalescence, higher strength, and ductility levels.

## Figures and Tables

**Table 1:** Compositions of polybutyrate-adipate-terephthalate–polymer (PBAT)/Wood flour blends.

Samples	PBAT100	PBAT95	PBAT90	PBAT80
PBAT (wt %)	100	95	90	80
Wood flour (wt %)	0	5	10	20

**Table 2:** Printing parameters.

Material	Extrusion Temp (°C)	Bed Temp (°C)	Spindle Velocity (RPM)	Print Velocity (mm/min)	Strand Gap (mm)	Air Gap (mm)		
						L1	L2	L3
PBAT100	120	110	1050	570	2.00	2.75	4.75	7.00
PBAT95	135	110	1100	570	2.25	3.00	5.00	7.00
PBAT90	130	110	1100	570	2.25	3.00	5.00	7.00
PBAT80	140	110	1300	570	2.60	2.50	4.25	6.25

**Table 3:** Dimensional variations of samples.

Material	Thickness (mm)		Width (mm)	
PBAT95	6.63	9.54%	23.33	14.25%
PBAT90	6.46	7.06%	23.51	14.93%
PBAT80	5.51	−1.19%	27.02	25.10%

**Table 4:** Mechanical properties of 3D printed (3DP) vs injection molded (IM) samples.

Sample	Strength (MPa)		Flexural Modulus (MPa)	
	3DP	IM	3DP	IM
PBAT95	53	49	403	483
PBAT90	39	42	345	558
PBAT80	31	39	418	767

**Table 5:** Flexural test results and mechanical properties.

Material	Force (N)	Maximum Deflection (mm)	Maximum Stress (MPa)	Maximum Strain	Flexural Modulus (MPa)
PBAT95	1440	2.8	53	0.1767	366
PBAT90	1030	2.4	39	0.1335	331
PBAT80	677	2.2	31	0.1274	423
